# Waking up every day in a body that is not yours: a qualitative research inquiry into the intersection between eating disorders and pregnancy

**DOI:** 10.1186/s12884-018-2105-6

**Published:** 2018-11-29

**Authors:** Elizabeth A. Claydon, Danielle M. Davidov, Keith J. Zullig, Christa L. Lilly, Lesley Cottrell, Stephanie C. Zerwas

**Affiliations:** 1 Department of Social & Behavioral Sciences, West Virginia University School of Public Health, Robert C. Byrd Health Sciences Center, West Virginia University, One Medical Center Drive, P.O. Box 9190, Morgantown, WV 26506-9190 USA; 2 Department of Biostatistics, West Virginia University School of Public Health, Morgantown, WV USA; 3 Department of Pediatrics, West Virginia University School of Medicine, Morgantown, WV USA; 4 Department of Psychiatry, UNC School of Medicine, Chapel Hill, NC USA

## Abstract

**Background:**

Women with eating disorders are more likely to negatively react to finding out they are pregnant, although this difference in attitudes between women with eating disorders and controls disappears at 18-weeks’ gestation. Those with anorexia also are twice as likely to have an unplanned pregnancy and those with bulimia have a 30-fold increased chance compared with healthy controls. Therefore, due to these considerations, pregnancy and the transition to motherhood can be an extremely challenging time for these women both psychologically and physically. The purpose of this qualitative descriptive study was to understand the intersection between eating disorders and pregnancy from the lived experience of women who have been pregnant or want to or do not want to become pregnant.

**Methods:**

A total of 15 women with a current or past history of an eating disorder were recruited, including nine women who have had previous pregnancies as well as six nonparous women. Interviews were the primary unit of data collection, in addition to document analysis of diaries or blogs. Data analysis was based on verbatim transcripts from audio recordings. NVIVO 11© was used to manage the data from these interviews and thematic analysis was then conducted for emergence of major and sub themes.

**Results:**

A total of six themes emerged from the iterative process of coding and categorizing. They were: *Control*, *Disclosure to Others*, *Battle between Mothering & Eating Disorder*, *Fear of Intergenerational Transmission*, *Weight and Body Image Concerns*, and *Coping Strategies*. One theme, *Battle between Mothering & Eating Disorder* also had three sub-themes: Decision to Have Child, Emotions Towards Pregnancy, and Focus on Child/Greater Good.

**Conclusions:**

It is hoped that quotes and themes derived from this study will help inform both prenatal and postnatal care and interventions, as well as addressing intergenerational transmission concerns among mothers with eating disorders.

**Electronic supplementary material:**

The online version of this article (10.1186/s12884-018-2105-6) contains supplementary material, which is available to authorized users.

## Background

“Waking up every day in a body that is not yours” is a quote that may resonate with many pregnant women, but for a pregnant woman with an eating disorder (ED) or history of an ED, this bodily disconnect is even more pronounced. Although they realize the bodily changes are necessary for the baby to grow, it is harder for women with, or history of, an ED to accept those changes. Instead, the weight and shape changes become constant triggers, making pregnancy a time of particular physical and psychological strain [1].

Women with EDs experience many battles throughout their disease, but considerably more when they face the prospect of pregnancy. Women with EDs are more likely to negatively react to finding out they are pregnant, although this difference in attitudes between women with EDs and controls disappears at 18-weeks’ gestation [2]. These negative reactions are thought to be due to several different factors [1]. First, women with anorexia nervosa (AN) are significantly younger at their first pregnancy than comparison groups, making them potentially less emotionally or developmentally prepared for the pregnancy [3, 4]. Another factor is that women with EDs are more likely than women without EDs to experience an unplanned pregnancy. Those with AN, for example, are twice as likely to have an unplanned pregnancy and those with bulimia nervosa (BN) have a 30-fold increased chance compared with healthy controls [5, 6]. It is still not completely clear why there are these considerably elevated rates of unplanned pregnancy among women with eating disorders, however one theory is that it could be due to patients’ belief that amenorrhea (cessation of menstruation) or oligomenorrhea (infrequent menstrual periods) are related to fertility and therefore indicate they cannot get pregnant [5]. Due to this mistaken belief of unlikely pregnancy, women with EDs may also be less likely to use adequate contraception. Therefore, with their negative attitudes towards pregnancy and higher risk of unplanned pregnancies, pregnancy and the transition to motherhood can be a traumatic time for these women both psychologically and physically. Understanding that process is critical to helping improve pregnancy outcomes and maternal-fetal bonding among these women, as well as helping improve and tailor prenatal care to their unique needs.

There is limited research on the intersection of EDs and pregnancy [1, 4–7] and very few studies are qualitative in nature [8, 9], which inhibits understanding this connection from a lived experience perspective. Two qualitative studies in particular built upon this gap but have limitations in describing the nature of this experience. For example, Clark et al. [8] described that women found pregnancy experiences (like feeling the baby kick) to be protective against body dissatisfaction during pregnancy, although that body positivity did not extend into postpartum. Tierney et al. [9] identified three types of women in their interviews: women who went into recovery during pregnancy and maintained that ED recovery postpartum, those that temporarily recovered during pregnancy, and those that were unable to give up ED behaviors during pregnancy. Both studies cited a need to be able to provide more information to women with EDs about expected body changes during pregnancy and to provide professional support to overcome issues of control that could inhibit a positive and healthy pregnancy. However, neither of the current qualitative studies compared participants across countries nor included participants from the United States; one was based in the United Kingdom [9] and the other in Australia [8]. Additionally, both studies also specifically included women who were or had been pregnant but did not look at women with EDs who were hoping to become pregnant 1 day, let alone address the concerns such women might have about becoming pregnant.

The purpose of this qualitative study is to describe the experience of pregnancy for women who have a current or past ED. A qualitative study is necessitated for this area of research because the topic is complex and requires participants’ perspectives and in-depth insight to truly understand their lived experiences. Only through such rich detail can we hope to understand the intersection between EDs and pregnancy from the experience of women who face those issues.

This study employed Qualitative Description [10]. This design is appropriate for gathering rich information about a topic where relatively little is known and then describing the phenomenon, process, or perspectives from the viewpoint of participants involved in their own language, without a highly theoretical or interpretive rendering of the data [11]. Therefore, interpretation of the data in Qualitative Descriptive research does not move far beyond what is provided to keep the findings closer to the original data [10]. The philosophical underpinning of this study was feminist theory, which addresses the various situations and institutions that women face, shaping their experience of the world [12]. The aim of most feminist theory approaches is to “correct both the invisibility and distortion of female experience in ways relevant to ending women’s unequal social position” [13]. A transformative framework also guided our description because it operates under the tenet that knowledge is not neutral but based on a complicated web of power dynamics that reflect social relationships and that therefore, must be used to improve society [14]. This transformative framework is aligned with the purpose of this study – to aid a marginalized population, specifically women with past or present EDs – in order to help people and thus improve society [15].

The specific aims of this study were to: 1) Describe the concerns that women with EDs feel in becoming pregnant, 2) Understand some of the unique barriers in prenatal care that women with EDs face and 3) Learn how women with EDs can be better supported throughout pregnancy to improve both maternal and child health outcomes.

## Methods

### Personal Lens

Qualitative research requires investigators to provide their own personal lens and background on research in order to check biases and ensure the trustworthiness of the data. This is a topic that the principal investigator (PI), EAC, is very passionate about on a personal level, having struggled with AN for several years. The PI is in recovery now after relapsing twice; however, during her last relapse, she became pregnant, which drastically changed her need and desire to take care of herself for the sake of her child. The PI received extensive support from doctors, psychiatrists, and nutritionists during her pregnancy, had a healthy pregnancy and subsequently, a healthy baby. However, the process of recovering from AN during pregnancy was incredibly challenging on an emotional level and the PI encountered many individuals who did not understand the pregnancy process from the viewpoint of a woman with an ED. Because of this experience, the PI felt it was imperative to understand this experience from women who have been pregnant and also the unique concerns that women have who want to (or do not want to) become pregnant and how their EDs affect these goals. The PI’s own experience is the reason for taking a transformative framework approach to this study because she understood the importance of translational research to change practice and prenatal care so that women with EDs are better supported during their pregnancies.

### Participants

An exemption was obtained from West Virginia University’s Institutional Review Board (IRB), the Office of Research Integrity and Compliance, negating the need for written consent because it was determined that a written consent would be the only documentation connecting them to the study (IRB #: 1602021245). Once that exemption was obtained, participants were recruited via personal referral and Facebook by asking for women (aged 18 and older) who have 1) an eating disorder, or a history of an eating disorder, and 2) have been pregnant or who did or did not want to become pregnant. Inclusion criteria were as follows: current eating disorder or history of an ED, past pregnancy (either planned or unplanned), or desire/lack of desire to have a pregnancy. Individuals who were currently pregnant were excluded due to ethical concerns about enrolling pregnant women with EDs who may be nutritionally deprived. An advertisement was also used in recruitment and all interested participants were provided a cover letter further explaining the study (See Additional file 1 for IRB-approved advertisement and cover letter).

Due to the PI’s history of an eating disorder and her transparency on social media about her history, she was well-positioned to have access to individuals who met the inclusionary criteria. Pseudonyms were used to protect the participants’ identities and were chosen using a random name generator. A total of 15 participants were interviewed. Eight participants who volunteered to participate were acquaintances or friends of the PI. Another friend acted as a gatekeeper to provide the PI with a total of five women who had been pregnant. Four of the five women were interviewed for this study; the fifth agreed to be in the study and was scheduled, but could not participate due to a family emergency, after which the PI was unable to get back in contact with her. Another co-author (SCZ) suggested two further participants: specifically, mothers who had blogged about their experiences of being pregnant with an ED. Therefore, this was a convenience sample, but purposive since individuals were specifically selected who could help to answer the research questions [16]. Additionally, all participants were made aware that the PI, who conducted all interviews, had a history of an ED and had been pregnant. At the time of the study, the PI had a Master of Public Health and a Master’s of Science degree and was completing her doctorate in public health; she also had training and expert guidance from a co-author (DMD) in qualitative research methods.

### Ethics and consent to participate

Participants were asked if the sessions could be audio-recorded and were assured that identifying information would be removed and would not be used in any reports or published materials. Any documents (such as diaries or blogs) were also de-identified before quotes were used. This study was filed with West Virginia University’s IRB, the Office of Research Integrity and Compliance, and was given an exemption for both interview data and document collection. A copy of the interview protocol including questions and probes can be found in the Additional file 1. A list of resources and referrals for ED treatment and support was created for the purpose of being provided to any women who became distressed during the interviews or who expressed interest in ED resources (see the Additional file 1). None of the participants became distressed, however one expressed a desire for more resources and another mentioned wanting to get treatment at some point; the resource and referral list was sent to both women.

### Data collection

Interviews were the primary unit of data collection, which is appropriate given that there was very sensitive and confidential information to be gathered that might not be as readily shared in a group format, such as through focus groups [16]. The PI conducted individual interviews with participants between March and August 2016, each lasting approximately a half-hour. These interviews were conducted by phone or Skype, depending on the participant’s convenience and preference. All interviews were conducted from the PI’s home, and, to the PI’s best knowledge, from the participants’ homes. No one else was present with the PI during the interviews, but sometimes a family member or child of an interviewee would be present momentarily on the interviewee’s side; the interview was stopped until the other person left. Participants were read a consent script and asked if they consented to participate before interview questions were asked. Participants also completed a one-page demographic form prior to the interview, in order to gather information regarding their age, highest level of education, current weight and height (if women did not feel comfortable providing their weight, they were not required to), number of children and year of first delivery, and current employment (See the Additional file 1). This form was sent out ahead of time to save time during the scheduled interview and to allow for the collection of some sensitive information such as weight that participants might feel more comfortable answering in writing.

Audio recording was conducted using QuickTime Player© for phone interviews and eCamm© for Skype interviews. Field notes were also taken throughout the interview on a printed interview protocol with information written under each pertinent question. Member-checking is a technique commonly used in qualitative research to improve the accuracy, credibility, and internal validity of a study and involves giving the informants the opportunity to check the authenticity of the material that is being collected [16]. In this study, member-checking was conducted by verifying information or repeating statements back to participants during interviews to check the validity of the information being captured. Member-checking was also conducted by circling back through email, or text message if preferred, with participants, post-interview, to clarify any areas that remained unclear.

Women who had been pregnant were asked to share diaries or blogs from the time when they were pregnant to help triangulate their interview data; a total of four participants volunteered to share pertinent diary or blog entries. Therefore, method triangulation was used and quotes from the diaries and blogs were also captured in this study through document analysis (consistent with triangulation recommendations from Creswell [16] to use “multiple and different sources, methods, investigators, and theories to provide corroborating evidence”; p. 251). We also employed source triangulation by drawing perspectives from women who had children as well as nulliparous women.

It is common for the interview protocol to change during qualitative research, which did occur to some extent during the course of this study. During the first two interviews, the topic of intergenerational transmission came up either in relation to the development of the participant’s ED or in their fear that their child/future child might have or develop an ED. Therefore, after those first two interviews, we included questions about intergenerational transmission as a probe if not mentioned or as follow up questions if mentioned.

### Data analysis

Data analysis was conducted in the form of thematic analysis, which is the act of gleaning major and subthemes from qualitative data [16]. The analysis was based on 15 verbatim transcripts from audio recordings; transcription of five interviews was conducted by the study’s PI using VideoLan’s VLC® (Version 2.2.2) multimedia player to slow the audio recordings. The other 10 interviews were transcribed using the transcription service Rev.com. A non-disclosure agreement was put into effect with this service due to the sensitive information within the interviews. All interviews transcribed by Rev.com were then reviewed by the PI while listening to the original audio content.

The coding of the transcripts followed a standard qualitative methodological process starting with an iterative transcript assessment and ending with theme development [16]. For example, each transcript was read post-transcription, and each one was iteratively read once another transcript was complete. In this way, and according to standard practice, transcripts were continually compared and contrasted between each other in order to assist with thematic analysis. Each transcript was also compared to the PI’s interview notes. After those initial readings, the PI went through each transcript and inductively assigned codes to sections of text that addressed a similar issue. NVIVO 11© was used facilitate all aspects of data management and coding. Some codes were in vivo using the guiding theories for the study and the rest were based on subjective assessment of the best encompassing descriptor [16]. Once all transcripts were coded, the codes on each transcript were reviewed in light of any new codes that may have emerged. This same process of coding was used after reviewing the transcripts for document analysis for the diaries and blogs.

All codes from the transcripts were tagged and then similar codes were grouped to create categories. Codes and categories that were repeatedly portrayed in transcripts then rose to the level of themes. Codes and categories were reviewed several times by the PI to ensure that codes and categories were collapsed or split as necessary into major themes. During that process – and according to standard qualitative research analysis – a few themes were again collapsed when it appeared that the lines between different themes blended better into one comprehensive theme [17].

A second coder also reviewed a total of four transcripts (26.7% of the sample), after training on the protocol, procedure, and coding system; this proportion of the transcripts is adequate according to Loewen & Plonsky [18] who recommend a second coder to code at least 20% of the sample. This second coder was also trained with two sample interviews prior to coding to ensure inter-rater reliability. Any discrepancies during training were discussed via multiple debriefing sessions and a final coding scheme was developed and agreed upon by both coders. After consensus was reached by both coders on the final coding strategy and structure, each coder used the agreed upon framework to code the remaining transcripts.

## Results

### Description of participants

Of the 15 participants interviewed, there were nine women with children and six nulliparous women. Participant demographics can be additionally seen in Tables 1 and 2, but the women will also be briefly described here. Ages listed are ages at time of interview. The nine mothers were named via the random name generator: Amelia, Nancy, Charlotte, Ann, Kathryn, Ruby, Nicole, Rose, and Laura. Amelia, age 27, is a mother of one, the result of an unplanned pregnancy at age 19. She continues to suffer from an Eating Disorder Not Otherwise Specified (EDNOS) – although EDNOS was the self-reported diagnosis, this diagnosis is now classified as ‘Other Specified Feeding or Eating Disorder’ (OSFED) in Diagnostic and Statistical Manual of Mental Disorders-5th Edition (DSM-5) [19]. Nancy, 38, is a mother of three, with a past history of AN (purging subtype); she was only just in recovery when she became pregnant with her first, at age 28. Charlotte, 30, suffers from BN, which impacted all of her three pregnancies, two of which were unplanned. Ann, age 55, has a history of AN, which influenced both of her pregnancies and continued for several years after her giving birth. Ann’s biological mother had AN, but Ann was raised by an adopted family from the age of 18 months. Kathryn, 31, also has a past diagnosis of AN, and had one child after being in recovery for several years. Ruby, also age 31, has a history of BN and had one child while in recovery although experienced some disordered eating symptoms throughout pregnancy. Nicole, 34, has a history of EDNOS, and became pregnant with her first child soon after coming out of inpatient treatment; her pregnancy, although not unplanned, was also not expected to occur so soon after. Rose, 32, had BN when she was much younger and had been in recovery for almost 20 years before having her first child. Laura, 41, also experienced AN when she was younger and had also been in recovery for about 20 years at the time of her pregnancy.

**Table 1:** Maternal demographic characteristics

Participant	Amelia	Nancy	Charlotte	Ann	Kathryn	Ruby	Nicole	Rose	Laura
Age	27	38	30	55	31	31	34	32	41
Relationship Status	In a relationship	Married	Married	Married	Married	Married	Married	Married	Divorced
Location	New York, U.S.	Virginia, U.S.	England, UK	California, US	Wisconsin, US	New Jersey, US	Georgia, US	Maryland, US	Massachusetts, US
Race	Slavic/White	Caucasian	White, British	Caucasian	White	White/Caucasian	Caucasian	Caucasian	Caucasian
ED Type	EDNOS^a^, current	Past AN (purging type)	Bulimia	Past AN	Past AN	Past BN	Past EDNOS	Past BN	Past AN
ED Active in Pregnancy	No	No	Partially	Yes	No	No	No	No	No
BMI	17.8	26.6	24.2	18.5	21.1	30.7	Height 5′9″, weight not provided	21.5	29
Education	High School	College	College	Graduate School	Graduate School	Graduate School	College	Graduate School	Graduate School
No. of Children	1	3	3	2	1	1	2	1	1
Child Sex	F	F, M, M	F, F, F	F, F	F	F	M, F	M	M
1st Child’s Birth Year	2009	2006	2005	1986	2014	2015	2012	2016	2013
Current Job	Pharmacy Tech	Digital Marketing Consultant	Not currently employed	Certified Psychotherapist	Research Associate (Postdoc)	Graduate Teaching Assistant	Executive Director	Biologist	Not currently employed

*AN* anorexia, *BN* bulimia, *BED* binge eating disorder, *EDNOS* eating disorder not otherwise specified

^a^Eating Disorder Not Otherwise Specified, now labeled ‘Other Specified Feeding or Eating Disorder’ in DSM-5

**Table 2:** Nulliparous demographic characteristics

Participant	Melissa	Maria	Marilyn	Kimberly	Sarah	Louise
Age	28	29	25	28	25	29
Relationship Status	Living w/partner but ending relationship	In a relationship	Single	Married	Single	Married
Location	Nova Scotia, Canada	San José, Costa Rica	Texas, US	Colorado, US	Delaware, US	TX, US
Race	Caucasian	Hispanic	Caucasian	White	Caucasian/white	Caucasian
ED Type	Bulimia, current	EDNOS, past	Past BED & current BN	Past AN & BN	Bulimia, current	Past AN & BED
BMI	20.8	22.3^a^	27.4	18.8	22.5	22.9
Highest Education	College	Graduate School	College	College	College	Medical School
Current Job	Support worker for individuals w/disabilities	Chief of Staff to Congressman	Paralegal	Romance Writer	Registered nurse	Medical Resident

*AN* anorexia, *BN* bulimia, *BED* binge eating disorder, *EDNOS* eating disorder not otherwise specified

^a^Since last weighing, but has not varied much

Six participants did not have children and had mixed feelings about future pregnancies due to their EDs; the six are: Melissa, Maria, Marilyn, Kimberly, Sarah, and Louise. Melissa, age 28, had a previous abortion from an unplanned pregnancy in 2014 and continues to suffer from BN. Maria, age 29, with a past history of EDNOS, had recently become engaged and expressed a desire to become pregnant after a few years of marriage. Marilyn, 25, has a history of binge eating disorder (BED) and was currently experiencing BN at the time of this study; due to her ED, she was not actively thinking of becoming pregnant, although theoretically considered surrogacy or adoption. Kimberly, 28, has a history of AN and BN, and does not want to have children, in part because of her ED history. Sarah, 25, has current BN, and is considering potentially adopting a child in the future. Louise, age 29, has a history of AN and BED, and is considering becoming pregnant in the next few years.

Due to the high rates of unplanned pregnancy in the ED population, it is also important to note the prevalence within this group of women. The women reported a total of 17 pregnancies, including two abortions; of those, six were unplanned, including those two abortions, and eleven pregnancies were planned.

### Thematic analysis

A total of six themes emerged from the iterative process of coding and categorizing. They were: *Control*, *Disclosure to Others*, *Battle Between Mothering & ED*, *Intergenerational Transmission*, *Weight and Body Image Concerns*, and *Coping Strategies*. One theme, *Battle Between Mothering & ED* also had three subthemes. A detailed description of these themes and subthemes can be found in Table 3.

**Table 3:** Themes/Subthemes

Theme/Subtheme	Description
Control	Mentions of control either regarding their eating disorder or pregnancy/future pregnancy
Disclosure to Others	Choice (& decisions in choice) to tell others about their ED, including family/friends or medical professionals
Battle Between Pregnancy & ED	The internal battle expressed between a woman’s ED & her mothering instinct
*Decision to Have Child*	*The factors involved in decided whether to have a future child or keep/abort an unplanned pregnancy*
*Emotions Towards Pregnancy*	*Emotions towards pregnancy or initial reactions upon becoming pregnant*
*Focus on Child/Greater Good*	*Focusing on the wellbeing of the child or sacrificing oneself for the ‘greater good’*
Intergenerational Transmission	Worry regarding whether their children or future children would also suffer from EDs or body image concerns
Weight & Body Image Concerns	Concerns about body image or weight primarily regarding pregnancy (including pregnancy weight gain & loss, as well as OB weight checks)
Coping Strategies	Ways expressed to cope with the challenges of a past or future pregnancy

### Control

Individuals with EDs often feel that the ED provides them with a sense of control. Typically, when other aspects of their life feel unmanageable, the ED with its behaviors and rules acts as their constant. It is not surprising perhaps, then, that control stood out as a major theme for these women, in terms of how they talked about their eating disorder and their view on pregnancy and the need to feel an aspect of control going into, or during, pregnancy.

Amelia spoke about the aspects of control that initiated and maintained her eating disorder and how being able to track everything helped her cope with the stress in her life. She also expressed how hard it was to have to give up that level of control because tracking her calories during pregnancy would, in her words, “completely drive me insane”:

That was the hardest for me, because at the end of the day, I didn’t have those numbers to look back on. I didn’t know how I did that day. I didn’t know like any of that so, it was a complete kind of loss of control and having to give that up because otherwise it was going to completely drive me insane. (Amelia, interview data, Spring 2016)

Melissa recounted her dread of not being in control of her body and how her belief that she could control her body by starving it was invalidated when she became pregnant unexpectedly.

I had that false, grandiose thought that I was in control of my body and I was calling the shots. And I wasn’t and it was kind of surprising. It was a humbling experience … I can’t just march ahead and not take any precautions thinking that I can just starve my body into compliance. (Melissa, interview data, 2016)

In contrast, Ann discussed trying to reassert control during her second pregnancy, since she felt she let others dictate what she did during her first pregnancy too much, which limited her ED behaviors and allowed her to gain more weight than she was comfortable with. Ann stated: “I am not doing whatever I did last time. No one’s going to tell me how much I can exercise. No one’s going to control me. I am never going in that body again.”

### Disclosure to others

The issue of maintaining secrecy or disclosing information about the disorder relates to the previous theme of control. The women discussed reasons why they kept their disorder secret or when and why they would disclose it to others. Melissa said: “I kind of just latched onto an eating disorder, ‘cause [sic] it was my best friend, my little secret, it was just mine.” Similarly, Amelia revealed:

I have never sought treatment and I am incredibly private about it and sort of like my one secret that I’ve always had and it’s sort of one of those things, like they can take everything else away from me, but they can never take this away. (Amelia, interview data, Spring 2016)

Even when women disclosed it to close others, such as their spouses, those partners still were not able to understand the depths of the problem, which still left the women feeling isolated. Ruby shared:

My husband has no conception of it. He knows a little bit about that I had an eating disorder and that I've yo-yo dieted and things like that, but he really doesn't understand the mental processes that go behind it, and the constantly being, I'm going to say obsessed. (Ruby, interview data, Summer 2016)

This secrecy or disclosure in relation to medical professionals was also pertinent for women who had been pregnant, as well as those who were contemplating a future pregnancy. The decision whether to discuss information about their eating disorder with an obstetrician or midwife was mixed based on their concerns and what they hoped to gain from sharing that information. Some voiced that it was important to be completely open whereas others, like Amelia, expressed: “I wanted no treatment, no therapy, nothing like that. My OBs are physical doctors … if I could physically take care of it myself, there was no need for me to involve them.” Ann also strongly agreed with that sentiment: “My doctor never knew any of this because I kept everything. Everything. No one knew anything. No one in the whole world. Only me.”

Meanwhile, Charlotte discussed some of the challenges that were involved in trying to disclose an ED to medical professionals and wanting treatment:

I certainly felt a lack of … communication between psychiatric care and maternity care and needing some sort of, it doesn't have to be a specialized midwife but just someone who can cross barriers and help you navigate your way through the pregnancy from both perspectives and not just one or the other. (Charlotte, interview data, Spring 2016)

### Battle between pregnancy & ED

One of the most predominant themes for all of the women was the battle between pregnancy and their ED. This battle played out in various different subthemes, including their decision whether to have a child (including whether to keep or abort an unplanned pregnancy), their emotions (both positive and negative) towards pregnancy, and their focus on the child or the greater good of sacrificing themselves or their ED behaviors for a child.

However, the most prevalent theme was the overall internal battle that these women expressed having when trying to balance the demands of pregnancy and mothering with their ED or history of an ED. Many adopted a ‘mama bear’ standpoint once they became pregnant, which would force them to change their behaviors, but others stated that being pregnant and having an ED or history of an ED could not be mutually exclusive and were bound to interact.

Those who had been pregnant recounted that battle vividly. Amelia stated: “I swear to god, I had like a countdown to when she was born, and I didn’t have to eat anymore.” And Charlotte expressed frustration because of “the guilt of not being able to get myself together for the sake of the baby.”

Ruby also mentioned how it was challenging not to use pregnancy as an excuse to engage in ED behaviors:

It becomes that battle where you're like well, I've got morning sickness. Should I use that as an excuse? I'm not going to say that I didn't do it once or twice, but it was mainly due to the fact that I was already throwing up anyway. (Ruby, interview data, Summer 2016)

Some dealt with this battle by figuratively disconnecting themselves from the pregnancy in order to push away the impact that their actions were having:

Rosie Robot was who carried the babies, but when you think about a robot carrying a baby, they don't have any emotion. There's nothing there. Then there was me who was trying to be me. I tried. I didn't know I was doing this stuff at that time. I had no idea. I never wore any maternity clothes, ever. I wouldn't wear maternity clothes. I hid it. I hid my pregnancy. Our next-door neighbors … didn't know I was pregnant until I was 9 months pregnant. (Ann, interview data, Summer 2016)

Even women who had not been pregnant speculated about their concerns of not being able to win this battle. For example, Kimberly expressed feeling: “so incredibly selfish … I feel like that kind of hidden darkness could be brought out by something like pregnancy, so I don’t know how that would work.”

#### Subtheme: Decision whether to have child

Although many women wanted to become pregnant and planned to do so, several women had unplanned pregnancies and ultimately made different decisions about whether to continue with their pregnancies. Marilyn, when asked – like the other nulliparous women about a potential future pregnancy – expressed thinking about adoption or surrogacy because of the effect of pregnancy on the body and her ED.

Melissa recounted her decision to have an abortion:

I just couldn’t do 9 months of that. Regardless of the after part, it was the process. So, I scheduled myself for a [dilation and curettage] D&C and had that procedure done I think the day before Christmas Eve and then went home for Christmas. (Melissa, interview data, Spring 2016)

Ann also had an abortion in college before having two later pregnancies that were planned and carried to term. “I just thought okay, I guess I’m having an abortion. I remember I scheduled my abortion on the day of a final. It was like I had a final at 11:00 and had the abortion at 2:00.”

Like Marilyn, Maria had considered surrogacy in the past during her ED, stating that: “I would rather carry my own kids. But at that time, I was … seriously considering it just because I didn’t trust myself enough I think to carry them.” Sarah agreed, suggesting that she was more in favor of adoption than carrying a child herself.

Kimberly’s decision not to have children was partially influenced by her ED history. “By the time I recovered I was well into my twenties and I just was so used to not wanting children that, you know, I didn’t start at that point.”

#### Subtheme: Emotions towards pregnancy

Although all women undergo a multitude of both positive and negative emotions towards pregnancies, especially unplanned pregnancies, these emotions can be even more fraught for women with EDs because of the personal struggles they face. The women with unplanned pregnancies described being scared, isolated or having an ‘earth-shattering’ moment upon finding out they were pregnant, whereas women with planned pregnancies expressed being delighted. Nulliparous women also weighed in on their emotions towards a theoretical future pregnancy.

Amelia, although, faced with an unplanned pregnancy, did recount in her personal diary at 5 months pregnant that she was “Getting kind of obsessed with the idea of being perfect. Perfect lover. Perfect wife. Perfect mother. Perfect anorexic.”

Melissa, however, had a different reaction to her unplanned pregnancy:

I’d missed a period, but I didn’t really think too much of that because … that was normal for me and so … I took a pregnancy test and it came up positive and it was probably the most earth-shattering moment of my life. (Melissa, interview data, Spring 2016)

Women generally reacted to planned pregnancies with positive attitudes, although some women talked about how their ED affected their responses to pregnancy even during planned pregnancies. For example, during her one planned pregnancy, Charlotte expressed the disconnect between wanting her child and the negative emotions of being unable to change her ED behavior: “She was just very much loved and wanted … even though I couldn’t help but go through the exact same pattern.”

Many nulliparous women had concerns about future pregnancies, which Kimberly voiced. “Yeah, I could see … the whole something being inside your body as a little weird to me. And it kind of gives me that same itchy feeling I would get when I’d like eat food I considered bad.”

#### Subtheme: Focus on child/greater good

Many pregnant women also turn their focus to their baby or make sacrifices for what is seen as the greater good. Even women who did not plan on becoming pregnant described needing to ‘bite the bullet’ and deal with the challenges for the sake of the baby. This focus on the child or the greater good was what helped or might help them come out of their EDs enough to be able to have healthy pregnancies.

Amelia used her ‘mama bear’ instinct to force herself to eat or gain weight for the sake of the baby. “You have to keep telling yourself over and over you know … this isn’t about you anymore like it’s never going to be about you again; it’s about this child.”

However, even women who had been in recovery for years had to use their focus on the child to help them through challenges in pregnancy associated with their history of an ED. Kathryn stated: “Eating disorder behaviors really just didn’t feel like an option. Kind of like a, ‘this isn’t about me, this is about someone else.’” Ruby agreed, saying that: “You don’t want to not eat because if you don’t eat then your baby’s not getting nutrition. It’s more than just about you at that stage.”

### Intergenerational transmission

All the women spoke about their concerns revolving around a theme of intergenerational transmission, which is defined here as the transmission of beliefs or norms around dieting, eating, and weight or shape across generations. This theme of intergenerational transmission reflected both the women’s recollection of parental comments or actions growing up or their concerns about how their comments or actions could influence their child or a potential child. For example, Amelia described being very careful about trying to create normalcy for her child, to not label foods as good or bad, and to not talk about her body negatively. Still, she was concerned by the body hyper-awareness that her daughter displayed at a young age and was also concerned that she was a hypocrite by promoting advice that she herself did not follow:

I think my hardest thing is I’m a total hypocrite, because I’m telling her you know, well as long as you are healthy, and you drink enough water and you get enough sleep, and you exercise and everything, you’re going to be fine … while at the same time, I don’t do that at all. (Amelia, interview data, Spring 2016)

Melissa expressed her worries about the health ramifications on her child, which influenced her decision to abort her pregnancy. She also felt that it was hard for her to be a support worker for those with disabilities when she had her own struggles:

It’s difficult to have an eating disorder and then be in the role that I’m in because I feel you know, it’s the imposter syndrome, right like how am I trying to guide these kids into healthy decision making when secretly I’m you know, I’m participating in my own kind of battle? (Melissa, interview data, Spring 2016)

Similarly, Maria divulged concern about having girls in a future pregnancy due to her history of an eating disorder, out of fear that they would be more likely than boys to also develop an eating disorder. “I’m sort of scared of having girls … I mean like it’s a stereotype because guys can also have eating disorders. But like I’m more scared of having girls like, being a mother to girls.”

Many women also shared how they felt they had been influenced by their parents’ behaviors or weight commentary.

I've done a lot of reading about this, I think part of the reason that I feel like I have a weight problem is because my mother had a weight problem, and I was never really able to accept myself as beautiful. Just from this body image perspective ... I never learned what was healthy. (Ruby, interview data, Summer 2016)

When I started dieting in middle school, she [Louise’s mother] actually taught me how to diet. She taught me how to count calories. She taught me how to weigh food, and how to prepare food, and encouraged me in dieting until it became a little more obvious that it was out of hand. I mean obviously, she didn’t mean for that to happen. (Louise, interview data, Summer 2016)

Some women also expressed confidence that they would know the warning signs if their child developed an eating disorder and would therefore be able to get them into treatment earlier. Ann discussed a more direct intervention of how her husband stepped in to try to break the cycle for their children:

Fortunately, my husband, when they were in second grade and third grade … because I was making like bizarre food. He completely took over food. He brought in chips. He normalized food for my girls. I think that probably saved them, considering that my own biological mother had an eating disorder and then I did. (Ann, interview data, Spring 2016)

### Weight & body concerns

Weight and body image concerns are central to all eating disorders and so it is understandable that they would feature as a central theme among concerns about pregnancy. A common theme arose that although the women described the weight gain as being for a positive reason, they still could not reconcile it with their fear of weight gain.

A few also mentioned concerns about how to lose the baby weight healthily without triggering their eating disorder again. Maria expressed that, “objectively I know that people gain weight in pregnancy, but I think another fear would be how I would lose it afterward … like how to do it in like the healthiest way possible.” Sarah affirmed this fear, stating that “feeling like you have to lose weight is definitely a trigger for an eating disorder.”

Weight checks with obstetricians during pregnancy were also an expressed fear, as well as how doctors would deliver information regarding weight gain. Some realized that being weighed at prenatal visits would be very stressful and thought about opting for, or did opt for, blind weighing. Maria thought she would choose that option, because she has chosen not to weigh herself and hoped that “somebody could know and do the checks, but it’s information that I’d rather not know.” However, the women differed in how they approached that experience. Kathryn was able to frame her weight checks positively, saying that “it didn’t feel as revealing the way I think being weighed when not pregnant does … it was kind of a relief … it didn’t really mean that I was a big, fat pig.”

Many women cited body image concerns as issues that they had or feared they would face during pregnancy. As Amelia’s body changed she expressed feeling like she was losing her body or felt trapped in her body. “I feel so fleshy, swollen, flabby, jiggly, sloppy, oily, nasty, just FAT. It’s fucking killing me. I miss restricting so badly … this body is like a prison and I want out.” ~ Amelia, personal diary, c. 5 months pregnant.

For Louise, projected body image issues included the expectation of the ‘ideal’ pregnant woman’s body and her concern that she would not conform to that ideal.

This is going to sound terrible, but I’m not going to be one of those girls who look pretty when they’re pregnant. They’ve got this nice slender body, and then they just have this little bump … No, mine’s going to be one of those like, “Well, I look like a marshmallow,” because that’s just how my body carries weight … I know that that’s how I’m going to look, and how I’m going to feel, and it scares me. (Louise, interview data, Summer 2016)

### Coping strategies

Both Amelia, during her pregnancy, and Maria when thinking about future pregnancy, noted some coping strategies that they did or would employ in order to help them through challenges.

Amelia particularly used distraction as a coping mechanism, as well as reframing the pregnancy as a “temporary medical condition.” “I completely threw myself relentlessly into school at that point … because I had to distract myself somehow.” She also let go of her accountability with tracking her eating and calorie intake:

I forced myself to not keep track of things anymore … because at the end of day I knew I had to be consuming at least 2300 calories and if I hit that, I’d be upset, but then if I didn’t hit that, I’d be upset. So, either way, you’re never going to win in that situation … I just had to give up accountability all together and just say fuck it. (Amelia, interview data, Spring 2016)

Maria, however, thought she would rely on professional and social support throughout pregnancy. She believed she would need this help because, she stated: “I don’t trust myself to make the best decisions regarding pregnancy. Maybe I would and I’m underestimating myself, but I would rather just have the outside support.”

Ruby attempted to avoid engaging in eating disorder behaviors, such as purging, through conscious effort and her knowledge that the behaviors were unhealthy*:*

I go to the bathroom at the end of the meal and it's like I have to remind myself not to do it. I choose not to because I know it's not healthy, but it's a choice that I make. (Ruby, interview data, Summer 2016)

## Discussion

From these conversations with women discussing the intersection between EDs and pregnancy, a total of seven themes rose to the forefront. Some, such as *Weight and Body Image Concerns* and *Control* were expected due to the nature of EDs. However, others like *Intergenerational Transmission* were not. The fact that this theme arose to salience supports the recent research that suggests social or environmental transmission of dieting behavior is something that individuals recall or are concerned about [20]. Additionally, it is critical to note that all these women expressed concerns about intergenerational transmission of their EDs or ED behaviors, even when the prospect of pregnancy was so frightening. For them to all raise this as a concern indicated that despite the *battle between pregnancy and their ED* that had existed or would exist, their focus on the well-being of the child won out.

The women’s expressed concern regarding *disclosure* to their doctors indicates an area of potential improvement within prenatal care, but also within the educational systems. Medical professionals could benefit from further training about EDs in order to help provide more sensitive information and care – particularly around weight and weight gain – to women who have or have had an ED. It also would be beneficial to explore these women’s *coping strategies* further to help provide psychological or social support on adjusting to pregnancy, especially since many pregnancies in women with EDs are unplanned.

Based on Tierney et al.’s [9] assessment of the three routes women with eating disorders take during and after pregnancy, we also grouped the women who had been pregnant into those categories. Research from large prospective studies shows that remission rates are 78% for purging disorder (PD); 34% for BN for full remission and an additional 29% for BN for partial remission [21]. However, there are no estimates available for AN because there are currently no clear criteria to establish maternal underweight during pregnancy. This study focused on women with a previous pregnancy who had current or past AN, BN, and EDNOS. Kathryn (past AN), Ruby (past BN), Nicole (past EDNOS), Rose (past BN), and Laura (past AN) all went into recovery or were in recovery when they became pregnant and maintained that recovery postpartum. Nancy (with AN purging subtype) and Amelia (with EDNOS) recovered during pregnancy and Amelia went back to ED behaviors immediately postpartum. Nancy was able to maintain her recovery after her first pregnancy and had a minor relapse after her second pregnancy before going back into recovery. Both Charlotte (BN) and Ann (past AN) continued ED behaviors during their pregnancies. Charlotte was able to stop her behaviors when she was 5–6 months pregnant during each of her three pregnancies, but never went into recovery, and went back to behaviors immediately after pregnancy. Ann continued her ED behaviors throughout both her pregnancies and did not go into recovery until years after.

### Strengths

The current study overcomes some of the limitations of previous qualitative studies by having a more international perspective, finding common themes across participants from the U.S., the United Kingdom, Canada, and Central America. Additionally, this study captures the experiences of childless women with EDs who did or did not want to become pregnant and how their eating disorder affected that choice. Those women who did have children also had a mixture of planned and unplanned pregnancies, providing a more nuanced understanding of those experiences.

Given the sensitive nature of these interviews and the personal details that interviewees were asked to share, conducting the interviews over the phone or via audio Skype actually may have facilitated sharing more personal information due to the increased distance between participant and interviewer.

Other strengths of this study were the steps taken to ensure its trustworthiness and internal validity [22]. For example, the method and source triangulation employed ensured credibility and confirmability of findings, as did member-checking to verify that transcripts were accurate and that the themes were representative. We used negative case analysis by including women who had not been pregnant; the fact that they highlighted similar concerns provides additional credibility. We provided a thick description of the methodology employed, allowing for the transferability of these findings. The dependability of the data was enhanced by using a second coder for over 20% of the sample and ensuring training for that coder. Additionally, we reached thematic saturation, reinforcing the internal validity of the selected themes.

### Limitations

There are several limitations that need to be acknowledged. First, there is limited method triangulation because only a few (*n* = 4) provided documents for document analysis, so primarily thematic analysis from interviews was used. However, source triangulation was ensured by using both nulliparous women with children, which adds to the trustworthiness of the study and its data. Second, the transferability of the study is limited because the sample is comprised entirely of Caucasians, with only one identifying as Hispanic. Therefore, we cannot know if the results would vary in different racial or ethnic groups. The majority of the women were also highly educated, so again, future studies would need to include different demographic groups to understand the transferability of the results. Finally, we used a qualitative descriptive approach for this study, given the dearth of existing information on the topic and small sample size. A more interpretive and in-depth approach to data analysis, such as Interpretive Description [23], may have allowed us to more fully explore several important themes and sub-themes that emerged in this analysis. For example, issues of “control” clearly emerged in this analysis, and as such we felt it rose to the level of a major theme; however, issues of control underpin EDs in general, and also may cut across or be represented in the other major themes presented here, such as coping strategies. For example, a pregnant woman could be afraid of control being taken away from her during the pregnancy and not disclose her ED to her obstetrician as a form of coping strategy. These preliminary findings lay an important foundation for future interpretive studies to clarify this issue with a larger, theoretically generated samples of women with EDs who have been pregnant, who wish to become pregnant, and those who do not want to become pregnant.

## Conclusions

The compelling insights on pregnancy gathered from women who suffer from EDs provide useful areas for future intervention to improve the prenatal, pregnancy, and postpartum period for similar women. Their concerns, challenges, and experiences resonate with each other despite the differences in their situations, lending credence that the themes gathered in this qualitative descriptive study may be generalizable to a larger group of women. Future research will need to explore both racial and cultural differences, as well as differences in socioeconomic backgrounds that were not as varied among these 15 women. Additionally, some women mentioned issues such as surrogacy or adoption that stemmed from their ED, which would be valuable to research further. One future area of research would be to more clearly understand what motherhood means to women with EDs or a history of an ED. Amelia discussed her “mama bear” mentality, but more research needs to be done to discern how others conceptualize what being a mother means.

For the PI, from a personal perspective, this research is both critically important, but also emotionally charged. Having experienced a pregnancy while in recovery from an ED, the PI could empathize with these women over their concerns and battles between the strength of their mothering instinct versus the power of their ED. Many of the women interviewed were appreciative of a study on this topic because they felt lost in a society that could not understand the battle that they had undergone or would 1 day face. Maria mentioned her worry that others would think some of her concerns superficial or vain. These are worries that women should not have to have. This research is playing a part in giving a voice to this population’s concerns in order to help them through such an emotionally and physically challenging process.

## Additional file

Additional file 1: Appendix. (DOCX 26 kb)

## Abbreviations

AN: Anorexia nervosa
BED: Binge eating disorder
BN: Bulimia nervosa
D&C: Dilation and curettage
DSM-5: Diagnostic and Statistical Manual of Mental Disorders-5th Edition
ED: Eating disorder
EDNOS: Eating disorder not otherwise specified
EDs: Eating disorders
IRB: Institutional review board
OSFED: Other specified feeding & eating disorder
