# Supplementary material for: Waking up every day in a body that is not yours: a qualitative research inquiry into the intersection between eating disorders and pregnancy
Source: BMC Pregnancy Childbirth. 2018 Nov 29;18:463. doi: 10.1186/s12884-018-2105-6 (PMC6267071; doi:10.1186/s12884-018-2105-6)
Supplement: Supplementary file 1 — Appendix. (DOCX 26 kb) [file 12884_2018_2105_MOESM1_ESM.docx]

# Appendix A

**IRB-Approved Advertisement**

West Virginia University

Pregnancy and Eating Disorders Study

(IRB: 1602021245)

Volunteers Wanted for Research Study

Eligible participants will be interviewed about their experiences or expectations about pregnancy and that relationship with their eating disorder/history of an eating disorder. Interviews will last no more than an hour and all information will be kept confidential.

**Eligibility criteria include**: English-speaking, women (age 18 and older) with a self-reported diagnosis of an eating disorder or past eating disorder, who have had a pregnancy, or want to become pregnant.

WVU IRB is on file

Please contact Dr. Danielle Davidov with any questions.

Dr. Danielle Davidov

Department of Emergency Medicine & Department of Social & Behavioral Sciences

[ddavidov@hsc.wvu.edu](mailto:ddavidov@hsc.wvu.edu) or (304) 293-4083

**Cover Letter**

Dear Participant,

This letter is a request for you to take part in an optional interview at your convenience about your perception and/or experience of pregnancy as a woman with an eating disorder. The purpose of this study is to better understand the intersection between eating disorders and pregnancy from the viewpoint of women with these disorders who have been pregnant, are pregnant, or want to or do not want to become pregnant. By understanding some of the unique challenges and barriers, as well as facilitators these women face, we can learn how to better help women with eating disorders during pregnancy, including helping to inform prenatal care, obstetricians, and midwives. The information you would provide is vital to helping other women who struggle with similar concerns.

This study is being conducted to fulfill requirements for a Qualitative Research Methods class project and is led by Elizabeth Claydon, MPH, MS who is a PhD student in the Department of Social and Behavioral Sciences at West Virginia University. This project is being overseen by her professor, Dr. Danielle Davidov, in the Departments of Emergency Medicine and Social and Behavioral Sciences.

If you agree to participate, you will be sent a short demographic form to complete. Then, Ms. Claydon will contact you to set up either a phone or Skype call to conduct a 45 minute to one-hour interview.

This interview will be kept completely confidential and all data will be de-identified. Your information will always only be identified by a study ID, never your name or any other identifying information. Your participation in this interview is completely voluntary and you may choose to withdraw at any time. This study has been filed with West Virginia University’s Institutional Review Board and approval has been obtained.

For purposes of this research, these interviews will be audio-recorded and then transcribed. Even after finishing the interview, if you do not feel comfortable with us keeping your data, you can ask that your audio-recording be deleted. No notes written by the interviewer during the interview will be kept either in that case.

Thank you so much for your time and willingness to help with this important project.

Sincerely,

Elizabeth Claydon, MPH, MS

#

# Appendix B

**Interview Protocol:**

**Time of interview:**

**Date:**

**Phone/Skype**

**Interviewee ID:**

**Description of the Project:**

The purpose of this study is to better understand the intersection between eating disorders and pregnancy from the viewpoint of women with these disorders who have been pregnant, are pregnant, or want to or do not want to become pregnant. By understanding some of the unique challenges and barriers, as well as facilitators these women face, we can learn how to better help women with eating disorders during pregnancy, including helping to inform prenatal care, obstetricians, and midwives. The information you provide today is vital to helping other women who struggle with similar concerns.

**Informed Consent:**

This interview will be kept completely confidential and all data will be de-identified. In order to ensure that any health information you share today cannot be linked back to you, if you do mention any identifying information in this interview it will be removed from any transcripts before the data is examined. Thus, your information will always only be identified by a study ID. Your participation in this interview is completely voluntary and you may choose to withdraw at any time. This study has been filed with West Virginia University’s Institutional Review Board and approval has been obtained. If you have any questions as we go through the interview today, please don’t hesitate to ask. If there are any questions that I ask that you do not feel comfortable answering, you are under no obligation to answer them.

For purposes of this research, these interviews will be audio-recorded and then transcribed. Even after finishing the interview, if you do not feel comfortable with us keeping your data, you can ask that your audio-recording be deleted. No notes written by the interviewer during the interview will be kept either in that case.

***Interviewer prompt:*** Do you have any questions before we begin the interview? (If no, then: Okay, let’s get started then. I am going to start recording now.

**Questions:**

***If participant is or has been pregnant, read questions labeled A; if participant has not been pregnant, read questions labeled B.***

A1) Let’s get started with a general discussion of your history with an eating disorder.

*Probe:* What type of eating disorder were you diagnosed with/have had?

*Probe:* Can you tell me about when this began and its progression?

A2) How would you describe the status of your eating disorder when you found out you were pregnant?

A3) What was initial reaction to finding out you were pregnant?

*Probe:* How did these feelings/reactions change over the course of your pregnancy?

*Probe:* Why, if at all, do you think these feelings changed?

A4) What was it like to have an eating disorder during your pregnancy?

*Probe:* Why do you think your eating disorder or eating behavior changed?

A5) What were some of the biggest challenges you faced during pregnancy?

A6) Can you describe what it was like to be weighed during your prenatal visits, given your history?

A7) Did you discuss your eating disorder/history of an eating disorder with your OB/midwife?

*Probe:* What did you tell your OB/midwife about your eating disorder/history of an eating disorder?

*Probe:* How would you have wanted them to change your care based on that knowledge?

A8) What challenges have you faced postpartum as a mother with an eating disorder/history of an eating disorder?

B1) Let’s get started with a general discussion of your history with an eating disorder.

*Probe:* What type of eating disorder were you diagnosed with/have had?

*Probe:* Can you tell me about when this began and its progression?

B2) As it relates to your eating disorder/history of an eating disorder, can you tell me about how you feel about pregnancy?

*Probe:* Can you describe how you think you might feel if you found out you were pregnant now?

*Probe:* What are some of the concerns you have about becoming pregnant?

B3) How, if at all, do you think your eating disorder would change during pregnancy? (What do you think it would be like to have an eating disorder during pregnancy?”)

*Probe:* for changes before and after finding out about potential pregnancy…

B4) What are your feelings about gaining weight with pregnancy and frequent weight-checks at the doctor during your pregnancy?

B5) If you become pregnant, is information about your eating disorder/history of an eating disorder something you would share with your OB/midwife?

*Probe:* If yes, what information about your eating disorder would you share with your OB/midwife?

*Probe:* If not, why would you not be comfortable sharing this information?

#

# Appendix C

REFERRAL LIST

Below is a list of Referrals, Helplines, & Support Groups

Inclusion on the list does not imply endorsement of these agencies or services. Payment for services rendered at these agencies is the sole responsibility of the patient and not the researchers of the current study.

**Referrals**

**Academy for Eating Disorders: Find a Professional**

<http://www.aedweb.org/index.php/education/eating-disorder-information-2>

**Helplines**

**National Eating Disorder Association free, confidential Helpline**

Monday – Thursday 9am-9pm, Friday 9am-5pm

**1-800-931-2237**

**Eating Disorders Association (UK)**

Adult Helpline: **011-44-8456-341414**

Youthline: **011-44-8456-347650**

Adult Helpline: (open 8:30 to 20:30 weekdays)

Youthline: (open 16:00 to 18:30 weekdays)

##### National Suicide Prevention Lifeline

**1-800-273-TALK** or **1-800-SUICIDE (784-2433)**

**Support Groups**

**Study of Anorexia & Bulimia (CSAB) Support Groups**

Family & Friends takes place on the 1st & 3rd Wednesdays of the month.

Pursuing Recovery takes place on the 2nd & 4th Wednesdays of the month.

**212.333.3444** ext. 256 or email: [emilyrosenthal@hotmail.com](mailto:emilyrosenthal@hotmail.com)

1841 Broadway (at 60th St)

4th Floor - Room 407

New York, NY 10023.

**Online Eating Disorder Support Group – “Lift the Shame”**

“Lift the Shame” is the first web-based support group of its kind specifically targeted to offer support and resources to pregnant women and moms with eating disorders. This support group is a free, confidential online group hosted by Jena Morrow, author, speaker and activist for eating disorders awareness, and Timberline Knolls Alumnae Coordinator.

**“Lift the Shame” is open to all and live on the web:**

3rd Sunday of each month | 6-7 p.m. Central | 7-8 p.m. eastern | 4-5 p.m. Pacific

**If you would like to participate, please register online at** [**www.timberlineknolls.org**](http://www.timberlineknolls.org/lift-the-shame-registration)

#

# Appendix D

**Demographic Information**

1. How old are you? Years

1. Are you currently ? (circle one)

**1 – Married**

**2 – Living with a partner**

**3 – Divorced**

**4 – Separated**

**5 – In a relationship**

**6 – Single**

1. What race do you consider yourself to be? .
2. What is your current height and weight (if you do not feel comfortable answering this, please leave blank: ft. in. lbs.
3. What is the highest year of school or college that you completed? (circle one)

**Grade School High School College/Vocational Professional/Graduate School**

1. How many children do you have? (please write 0 if none)

**children**

1. What year did you have your first child? .
2. Are you currently employed?

**No**

**Yes**

If yes, what is your job title? .
